# Decreased *In Vitro* Artemisinin Sensitivity of *Plasmodium falciparum* across India

**DOI:** 10.1128/AAC.00101-19

**Published:** 2019-09-23

**Authors:** Rimi Chakrabarti, John White, Prasad H. Babar, Shiva Kumar, Devaraja Gouda Mudeppa, Anjali Mascarenhas, Ligia Pereira, Rashmi Dash, Jennifer N. Maki, Ambika Sharma, Kabita Gogoi, Devojit K. Sarma, Ipsita Pal Bhowmick, Suresh Kumar Manoharan, Edwin Gomes, Jagadish Mahanta, Pradyumna Kishore Mohapatra, Laura Chery, Pradipsinh K. Rathod

**Affiliations:** a Department of Chemistry, University of Washington, Seattle, Washington, USA; b Department of Global Health, University of Washington, Seattle, Washington, USA; c Department of Medicine, Goa Medical College and Hospital, Bambolim, Goa, India; d Regional Medical Research Center—Northeast Region (RMRC-NE), Dibrugarh, Assam, India

**Keywords:** Indian *Plasmodium falciparum*, RSA, artemisinin sensitivity, *kelch*

## Abstract

Artemisinin-based combination therapy (ACT) has been used to treat uncomplicated Plasmodium falciparum infections in India since 2004. Since 2008, a decrease in artemisinin effectiveness has been seen throughout the Greater Mekong Subregion. The geographic proximity and ecological similarities of northeastern India to Southeast Asia may differentially affect the long-term management and sustainability of ACT in India. In order to collect baseline data on variations in ACT sensitivity in Indian parasites, 12 P. falciparum isolates from northeast India and 10 isolates from southwest India were studied *in vitro*. Ring-stage survival assay (RSA) showed reduced sensitivity to dihydroartemisinin in 50% of the samples collected in northeast India in 2014 and 2015. Two of the 10 assayed samples from the southwest region of India from as far back as 2012 also showed decreased sensitivity to artemisinin. In both these regions, *kelch* gene sequences were not predictive of reduced artemisinin sensitivity, as measured by RSA. The present data justify future investments in integrated approaches involving clinical follow-up studies, *in vitro* survival assays, and molecular markers for tracking potential changes in the effectiveness of artemisinin against P. falciparum throughout India.

## Abstract (precis)

Artemisinin-based combination therapy (ACT) has been used to treat uncomplicated Plasmodium falciparum infections in India since 2004. Since 2008, a decrease in artemisinin effectiveness has been seen throughout the Greater Mekong Subregion. The geographic proximity and ecological similarities of northeastern India to Southeast Asia may differentially affect the long-term management and sustainability of ACT in India.

## INTRODUCTION

Artemisinin-based combination therapy (ACT) has been the first line of treatment for Plasmodium falciparum infection in India since 2004 and has been considered effective. However, the emergence of artemisinin resistance in some parts of the Greater Mekong Subregion poses a potential threat to this status (1). The northeast (NE) Indian states of Arunachal Pradesh, Nagaland, Manipur, and Mizoram share a 1,126-km border with China in the north and a 1,643-km border with Myanmar to the east (2, 3). These Indian states have overlapping ecologies with Southeast Asia, including similar topography, rainfall pattern, and vector species, that are different from those in the rest of India. Both China and Myanmar, along with the other Southeast Asian countries of Cambodia, Thailand, Vietnam, and Laos, have reported the slow clearing of parasite isolates after artemisinin treatment (1, 4–11), which is considered an indicator of decreasing artemisinin effectiveness (12, 13).

While this manuscript was in preparation, a clinical study in the state of West Bengal reported the first known instance of delayed parasite clearance after ACT treatment in India, with the parasites showing a higher survival rate in the ring-stage survival assay (RSA_0–3hr_) (14, 15). The study confirms that decreased artemisinin sensitivity now exists in eastern India; however, the distribution and magnitude of such an effect remain unknown (16–19). A continuing challenge for India is to determine whether the decreased effectiveness of the artesunate-sulfadoxine-pyrimethamine (AS-SP) regimen is restricted to the east and northeast regions or whether it can also be found in other parts of the country and if reduced effectiveness exists against other ACT regimens.

The gold standard of ACT resistance is delayed *in vivo* parasite clearance after drug treatment, ideally assessed by 42 or 63 days of clinical follow-up. Such longitudinal clinical evaluations can be challenging. In this respect, the method of *in vitro* RSA (RSA_0–3hr_) (20) offers some advantages; a sample drawn at a single time point per patient and cryopreserved can be tested *in vitro*, at a later time, in a specialized malaria lab (18).

In addition to high RSA_0–3hr_ values, some mutations in P. falciparum *kelch* (*Pfkelch*; Pf3D7_1343700) have been associated with decreased *in vivo* parasite clearance rates (20, 21). Previously published sequence data for Indian isolates in the east and northeast regions show mutations in the *kelch* gene; however, the ability of *kelch* mutations to predict decreased ACT efficacy in Indian parasites has been inconsistent (22–24).

In the present study, clinical P. falciparum samples from two geographically distant settings in India, one in southwest (SW) India (Goa State) and one in northeast India (Assam, Arunachal Pradesh, and Tripura States), were studied. Artemisinin sensitivity was assessed *in vitro* using RSA_0–3hr_, and parasite DNA sequences at the *kelch* locus were studied.

(This work was undertaken as a part of the Malaria Evolution in South Asia [MESA] program, a U.S. National Institutes of Health [NIH]-funded International Center of Excellence for Malaria Research [ICEMR].)

## RESULTS

### Demography and clinical history of Indian samples.

**FIG 1:**
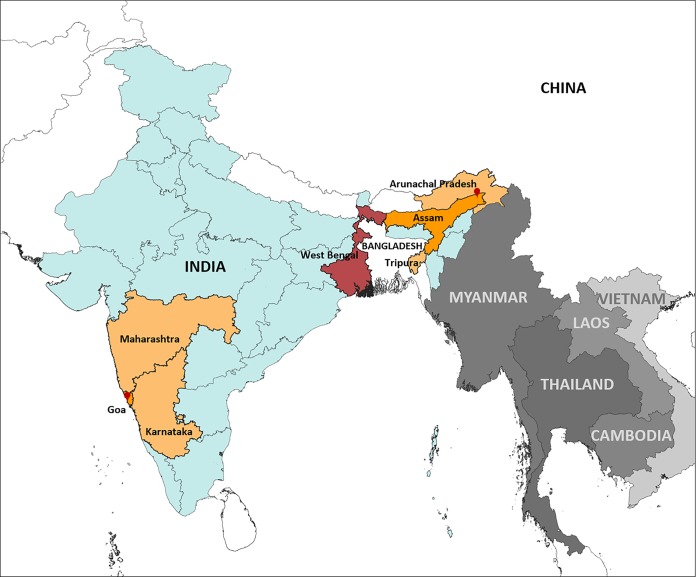
Map denoting the states of origin of the parasite isolates and the locations of the study sites in the northeast and southwest regions of India. The isolates from the northeast and southwest regions of India were collected and analyzed at the study sites at the Regional Medical Research Center (RMRC) in Dibrugarh and Assam and at the Goa Medical College and Hospital (GMC) in Bambolim, Goa, respectively. A recently published clinical study (15) reported decreased artemisinin sensitivity in West Bengal in the eastern part of India.

The samples from northeast India came from the border states of Assam, Arunachal Pradesh, and Tripura. The samples from southwest India were collected in the state of Goa (Fig. 1 and Table 1). The demographics of local inhabitants from Assam, Arunachal Pradesh, and Tripura constitute the demographics of northeast India, whereas the samples from the southwest were collected from residents of Goa as well as individuals who had recently moved from the neighboring states of Karnataka and Maharashtra to Goa. In both regions, northeast and southwest, transmission undergoes a seasonal peak during the monsoons and continues at a reduced intensity throughout the year.

**TABLE 1:** Demography, clinical history, and artemisinin sensitivity of the Indian parasite isolates used in the present study^*a*^

Parasite line	Enrollment date	Gender	Age (yr)	State of residence^*b*^	Day 0 parasitemia (%)	Parasite density (no./μl)	Severe malaria (degree of severity)	Treatment regimen^*c*^	Inpatient or outpatient (no. of days in hospital)	RSA_0–3hr_ survival rate^*d*^ (%)	*kelch* mutation
SW1	Apr 2012	Male	38	Karnataka	0.3	ND	No	AS, MQ	In (ND)	0.35	WT
SW3	Jun 2012	Male	19	Goa	0.5	1,579	No	AS, MQ (41.2)	In (5)	0.21	WT
SW24	Aug 2012	Female	37	Goa	1.3	8,238	Yes (1)	AS, MQ	In (8)	0.4	Insertion
SW25	Aug 2012	Male	60	Goa	2	8,078	Yes (2)	AS	In (7)	1.43	WT
SW31	Aug 2012	Male	17	Goa	0.6	4,360	No	AS, MQ	Out	0.28	WT
SW37	Aug 2012	Male	60	Maharashtra	1.9	3,200	Yes (5)	AS, PQ	In (10)	2.72	WT
SW45	Aug 2012	Male	35	Goa	0.1	660	Yes (1)	AS, MQ	In (8)	0.66	WT
SW46	Aug 2012	Male	40	Goa	1.9	205,005	No	AS, MQ, PQ	Out	0.36	Insertion
SW81	Sept 2012	Male	15	Goa	2.4	3,542	No	AS, MQ, PQ	Out	0.28	WT
SW136	Oct 2012	Male	26	Goa	1.9	5,372	No	PQ, CQ	Out	1.8	WT
NE10	Oct 2014	Male	34	Assam	2.8	5,600	Yes (2)	AS	In (1)	1.13	Insertion
NE16	Oct 2014	Male	20	Assam	11	260,000	No	AS, QN	In (4)	0.21	Insertion
NE17	Nov 2014	Male	32	Assam	0.2	6,640	No	AS, PQ (0.5)	In (5)	7.99	WT
NE20	Jan 2015	Male	35	Assam	42	368,880	No	AM (24)	In (2)	8.3	Insertion
NE27	Jun 2015	Female	35	Assam	47	684,000	Yes (1)	AS	In (2)	3.19	Insertion
NE28	Jun 2015	Male	19	Assam	0.8	16,037	Yes (3)	AS, LF	In (10)	0.3	WT
NE33	Jul 2015	Male	18	Arunachal Pradesh	42.6	1,648,400	Yes (1)	AS	In (5)	2.88	Insertion
NE38	Nov 2014	Male	40	Arunachal Pradesh	0.9	54,400	No	AM, LF, PQ	Out	0.59	WT
NE39	Nov 2014	Female	5	Arunachal Pradesh	0.1	2,520	No	AM, LF, PQ	Out	1.4	Insertion
NE45	Dec 2014	Female	2	Tripura	0.3	9,840	No	AS, PQ	In (2)	3.77	Insertion
NE46	Dec 2014	Male	4	Tripura	4.2	79,840	No	AS, PQ	In (2)	0.18	WT
NE53	Oct 2015	Female	14	Assam	2.2	83,400	No	AS, PQ (15.2)	In (5)	2.01	Insertion, A675V

a AS, artesunate; AM, artemether; In, inpatient; LF, lumefantrine; MQ, mefloquine; ND, not determined; Out, outpatient; PQ, primaquine; QN, quinine; WT, wild type.

b The state of residence was determined to be the place where the patient had stayed for at least 3 weeks prior to the blood draw.

c Values in parentheses represent the duration of treatment (in hours) before the blood draw, which applies to some patients who were treated before samples were collected.

d The RSA_0–3hr_ survival rate for each sample was evaluated by two microscopists, and the data in the table represent the average of two counts.

Culture-adapted samples from the northeast (NE; *n* = 12) and southwest (SW; *n* = 10) were used for RSA (Table 1). Both study groups (NE and SW) had similar proportions of severe malaria cases. The treatment regimen at the time of sample collection comprised artemether-lumefantrine (AM-LF) in northeast India and artesunate-mefloquine (AS-MQ) in southwest India. The AS-MQ regimen prescribed in the Goa Medical College and Hospital (GMC) during the study period was different from the standard artesunate-sulfadoxine-pyrimethamine (AS-SP) regimen used in all parts of India except the northeast states (25). The ACT regimen in the northeast states was changed from AS-SP to AM-LF in 2013 (25) due to the sulfadoxine-pyrimethamine (SP) resistance reported in this region (26–28).

### Classification of morphological changes in the DHA-treated parasites.

**FIG 2:**
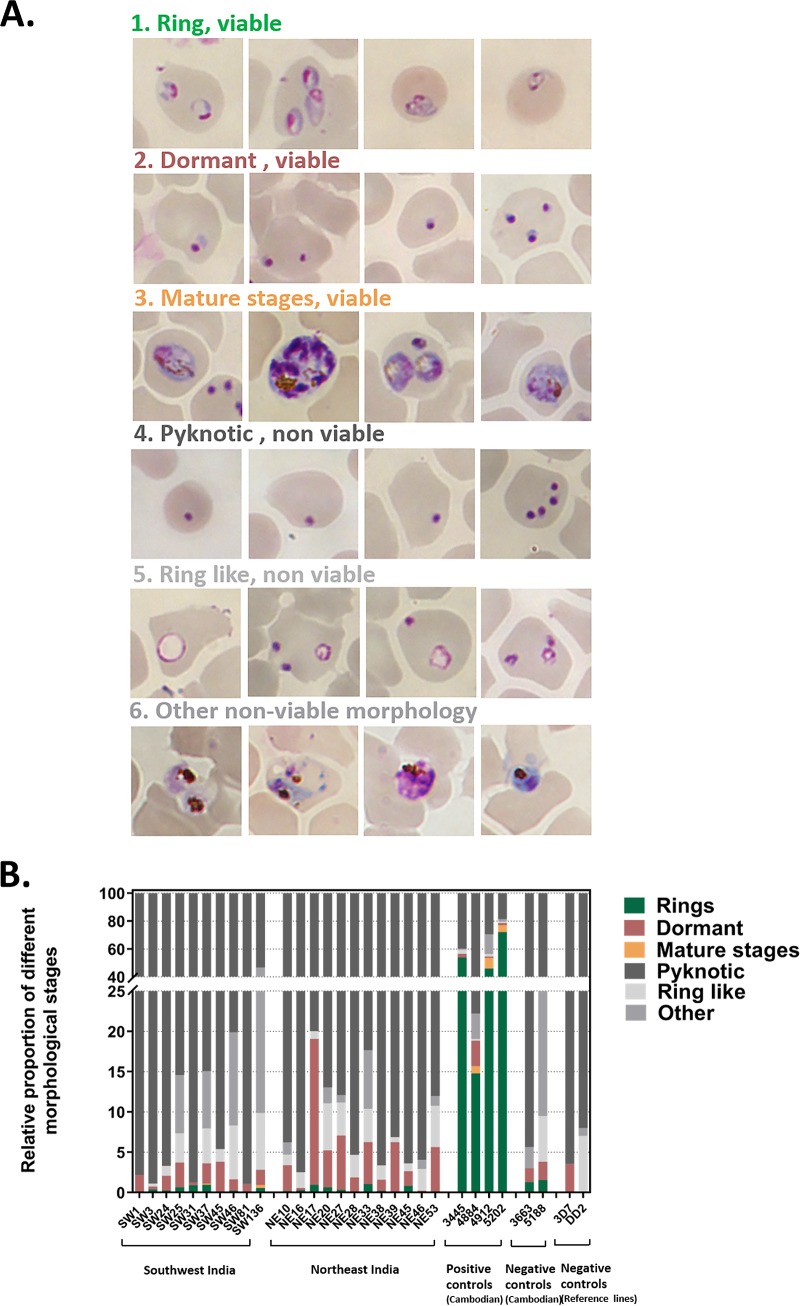
Phenotypes of dihydroartemisinin (DHA)-treated Indian isolates. (A) Morphology of DHA-treated parasites at the end of RSA_0–3hr_. The viable population comprised typical rings (row 1) and mature stages (row 3), but also included quiescent rings known to be dormant (row 2). Nonviable parasites, on the other hand, exhibited a pyknotic morphology, and some had ring-like structures, while others were extracellular or devoid of regular cytoplasm. (B) The relative proportion of different morphological stages in the treated parasite population show that a higher proportion of viable parasites was found among the isolates from the northeast than among the isolates from the southwest. However, both these groups had a much lower proportion of viable parasites than the positive controls (Cambodian artemisinin-resistant isolates).

Ring-stage parasites from southwest and northeast India treated with dihydroartemisinin (DHA) for 6 h exhibited distinct phenotypes (Fig. 2A). Viable rings, along with parasites that displayed an arrested ring morphology, were observed. The arrested ring-stage parasites had a round morphology with distinct dark red/pink-stained chromatin and a light blue-stained cytoplasm, similar to the findings for dormant parasites described in previous reports (29, 30). The majority of treated parasites were pyknotic, as defined by collapsed nuclei that stained dark purplish red with Giemsa and no significant cytoplasm. Other nonviable parasites showed a ring-like chromatin morphology but lacked a regular cytoplasm. Parasites with another deformed phenotype included parasites that were morphologically similar to mature stages but that lacked a distinct chromatin organization. Viable parasites in the DHA-exposed cultures exhibited an arrested ring or dormant morphology, along with small populations of viable rings (Fig. 2B). The proportion of viable parasites among DHA-treated southwest isolates (median = 2.1%) was lower than that among the Cambodian resistant lines (median = 36.7%). Within the two Indian groups, the northeast isolates, on average, had a higher proportion of viable parasites (median = 4.3%).

### Ring-stage survival after DHA exposure.

**FIG 3:**
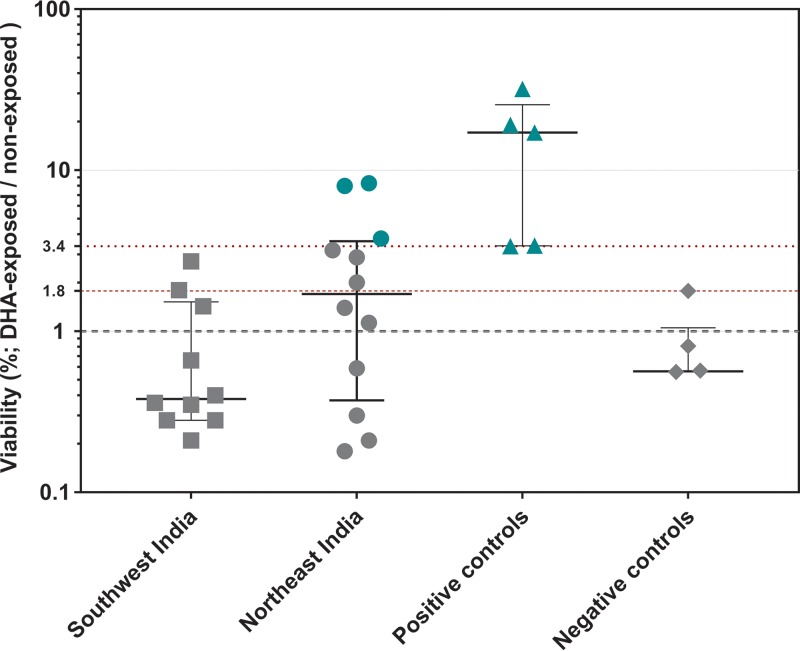
RSA_0–3hr_ survival rates of isolates from northeast and southwest India. The survival rate was calculated as the percentage of viable Plasmodium falciparum parasites in 700 nM dihydroartemisinin-treated test samples compared to the number in the dimethyl sulfoxide (DMSO)-treated controls at the end of 72 h of RSA. The horizontal bold lines represent the medians, and the whiskers at each end identify the interquartile range (IQR). Positive controls included clinically verified artemisinin-resistant Cambodian lines (IPC 3445, 4884, 4912, 5202), and negative controls comprised clinically verified artemisinin-sensitive Cambodian lines (IPC 3663, 5188), as well as laboratory reference strains 3D7 and Dd2. The four dotted lines represent the conventional RSA threshold of 1% along with the empirical thresholds obtained in this study at 1.8% (100th percentile for the negative controls) and 3.4% (0th percentile for the positive controls). Indian isolates and Cambodian artemisinin-resistant lines for which the results are above 3.4% are marked in green and denote the most conservative estimate of artemisinin-tolerant Indian isolates in this study.

The tolerance to artemisinin in the cultures exposed to DHA for 72 h varied considerably between the two groups of Indian parasites (Fig. 3). The RSA_0–3hr_ survival rate of parasites from the northeast (median, 1.7%) was three times higher than that of parasites from the southwest (median, 0.4%). In total, 8 of 12 (66%) northeast isolates and 3 of 10 (30%) southwest isolates had a survival rate of greater than 1%. The 1% or higher RSA_0–3hr_ survival rate is considered indicative of artemisinin tolerance (10). Both Indian groups had a lower survival rate than the artemisinin-resistant controls from Cambodia obtained from BEI Resources (median survival rate, 17.1%; range, 3.3% to 31.9%). Parasite strains in the negative-control group (artemisinin-sensitive strains) had a survival rate range between 0% and 1.7%, with the median survival rate being 0.5%. This group comprised the BEI Resources artemisinin-sensitive Cambodian strains and standard laboratory reference lines 3D7 and Dd2.

Compared to the survival rates for the controls, three northeast Indian isolates had survival rates above 3.4%, which was the 0th percentile (P0) or lowest survival rate of the Cambodian resistant strains. No southwest Indian isolate reached this threshold. Six northeast isolates and two southwest isolates had survival rates higher than 1.8%, the 100th percentile (P100) for the negative controls. The Cambodian strains with verified artemisinin resistance status served not only as reference points for survival rate comparison but also as internal controls for the RSA experiment itself under our test conditions. A statistically significant difference (*P* = 0.0025, Kruskal-Wallis test for the location of sample collection variable) was noted between the three groups of northeast and southwest Indian isolates and the Cambodian positive controls. The southwest isolates were statistically significantly different from both the northeast isolates (*P* = 0.0208) and the Cambodian artemisinin-resistant strains (*P* = 0.0002). In comparison, northeast isolates and resistant strains from Cambodia were statistically significantly similar (*P* = 0.0994). Statistical analysis of the RSA_0–3hr_ survival rate by two microscopists showed good agreement (Pearson correlation *r* = 0.75, *P* < 0.0001).

### Molecular characterization of *kelch* from northeast and southwest Indian isolates.

**FIG 4:**
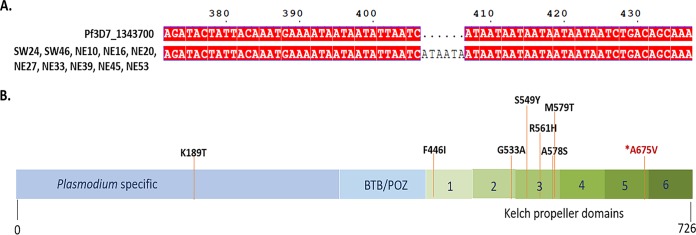
K13 mutations in Indian isolates. (A) Alignment of the nucleotide sequences of the *Pfkelch* gene (Pf3D7_1343700; https://plasmodb.org/plasmo/app/record/gene/PF3D7_1343700) from Indian isolates compared to the reference sequence from 3D7. Sequencing data indicated an insertion of 6 nucleotides in isolates SW24, SW46, NE10, NE16, NE20, NE27, NE33, NE39, NE45, and NE53. (B) Position of the single nucleotide change in NE53. The resulting nonsynonymous mutation in NE53, A675V (marked in red), is in the propeller domain of the Kelch protein. Nonsynonymous mutations marked in black have been previously reported from Indian patient samples but were not seen in the present collection.

DNA sequencing of the Indian isolates revealed two types of mutations in the *kelch* gene, a common insertion and a nonsynonymous mutation. The insertion of 6 nucleotides (coding for Asn-Asn [NN]) was observed in patient isolates SW24 and SW46, as well as patient isolates NE10, NE16, NE20, NE27, NE33, NE39, NE45, and NE53, at nucleotide position 407 (between codons 142 and 143) in the BTB/POZ (broad-complex, Tramtrack, and Bric-à-brac/poxvirus and zinc finger) domain of Kelch (Fig. 4A) (GenBank accession number MK949521). The contribution of this insertion to artemisinin resistance is unknown. However, it is notable that this insertion was relatively frequent in Indian isolates (unpublished data). It was more prevalent in samples from northeast India (66%) than in those from southwest India (20%). Out of 11 samples showing a >1% survival rate, 7 (63%) had this insertion. Among the 10 samples that had the insertion, 3 (30%) had a survival rate lower than 1%.

The only nonsynonymous mutation in the Kelch propeller domain was noted in patient isolate NE53, in which an alanine-to-valine mutation at position 675 (A675V) was seen (Fig. 4B) (GenBank accession number MK949522). The corresponding RSA survival rate was 2%.

## DISCUSSION

### Altered artemisinin sensitivity in Indian P. falciparum isolates.

The RSA_0–3hr_ survival rate of Indian isolates varied widely (0.2% to 8.1%), reflecting vastly different *in vitro* sensitivities to artemisinin (Table 1). These survival rates were similar to recently reported survival rates from a treatment study in east India (14, 15) and between the survival rates of Southeast Asian and African isolates reported elsewhere (10, 20, 31–33). None of the Indian groups exhibited consistently high survival rates like the isolates from Pailin, Cambodia (median, 14.9%), where artemisinin resistance is entrenched (10). However, they also do not resemble the survival rates of Ugandan and Gambian isolates from the same period when the Indian isolates were recovered. The Ugandan and Gambian isolates from that period showed consistently low survival rates (0% to 1%) (31, 34), though two very recent studies have reported elevated RSA_0–3hr_ survival rates (0.2% to 34.3%) in African parasites (32, 35). In the future, recording of *in vivo* parasite clearance data for Indian and African isolates, alongside *in vitro* RSA survival data, will further help define the best ways to assess the status of artemisinin sensitivity in these regions.

The RSA_0–3hr_ survival rate has been a reliable *in vitro* indicator of the clinical correlate of artemisinin resistance measured by parasite clearance after ACT treatment (10, 36). The RSA_0–3hr_ threshold of 1% was originally promoted on the basis of the corresponding parasite clearance half-life data for Cambodian isolates. Isolates with a clearance half-life below the threshold of 5 h were considered fast clearing, and those with a clearance half-life above that threshold were considered slow clearing (20). A 1% survival rate has since been adopted as the RSA threshold in subsequent *in vitro* resistance studies across different study sites in Southeast Asia and Africa. With an initial 77% accuracy rate in identifying slowly clearing parasites (10), the 1% threshold can capture the bulk of resistant isolates. In the absence of large-scale parasite clearance data collected from every region, which is needed in India, it seemed logical to apply a second, empirically derived threshold level to obtain a more conservative estimate of resistant isolates.

Two empirical thresholds were considered for the current study: the first was the 100th percentile (P100) for the negative control, which considers the highest RSA value for negative controls (1.8%) to be the threshold. The second threshold considered was the 0th percentile (P0) for positive controls, i.e., the lowest RSA value for positive controls (3.4%). Considering these most conservative estimates of threshold values, at least 3 northeast Indian isolates (NE17, NE20, and NE45) out of a total of 22 (13%) could be categorized as having a decreased artemisinin sensitivity phenotype. RSA_0–3hr_ survival rate cutoffs like these, based on actual experimental data, may also be a better indicator of region-specific artemisinin tolerance trends than the generic 1% threshold.

### Decreasing artemisinin sensitivity in India without *kelch* mutations.

The single nonsynonymous mutation, A675V, found in Kelch in the isolate from one patient in this study has been considered a candidate marker for artemisinin resistance by WHO (1). It is reported here from India for the first time. A675V was previously seen in six different areas of the Greater Mekong Subregion and in east Africa: on the Indonesia-Myanmar border (37), in southern Myanmar (38), at the China-Myanmar and Thailand-Myanmar borders (33, 39), in Rwanda (40), and in Uganda (35). The mutation was associated with slowly clearing clinical infections in Thailand (39) and delayed *in vitro* parasite clearance in Southeast Asian samples (30). However, A675V was also found in an artemisinin-sensitive clinical sample in Myanmar (38), suggesting that this mutation in itself cannot be the sole cause of artemisinin tolerance.

In the current study from India, no significant association between the RSA_0–3hr_ rate survival and *kelch* mutations was observed (Table 1). The only Kelch mutation seen, A675V in sample NE53, was associated with a 2% survival rate. However, for all other samples with a >1% RSA_0–3hr_ survival rate, there were no *kelch* mutations. Interestingly, such discordance (albeit in a smaller proportion) was noted in culture-adapted western Cambodian isolates, with *kelch* mutations being absent in about 11% of the isolates exhibiting a >1% RSA_0–3hr_ survival rate (41).

The Asn-Asn (NN) insert between codons 142 and 143 of *kelch* reported here is the first of its kind seen in India (Table 1). However, previous studies have reported one or two NN insertions at codon 142 of *kelch* in isolates from Senegal (42) and in Cambodia (41) and between codons 136 and 137 of *kelch* in isolates recovered on the China-Myanmar border (43). The NN *kelch* insert between codons 142 and 143 was observed in 45% of the Indian samples, and it was most prevalent in the northeast isolates (66%). Within the northeast India group of isolates, the NN insert was associated with seven out of eight samples with a >1% RSA survival rate. None of the southwest India samples with this insert (20%) had a >1% survival rate. Although these findings are interesting, on the basis of the number of samples with the NN insert between codons 142 and 143 in this study, it is not possible to infer any association of this insert with changes in artemisinin sensitivity.

Overall, the molecular data presented in this study from India and the historical prevalence of *kelch* polymorphisms suggest that the effectiveness of the *kelch* locus as a marker for artemisinin resistance surveillance, by itself, is not robust. This is backed by observations in western Myanmar, where the proportion of samples positive on day 3 was much less than the proportion of isolates with K13 propeller mutations (37). Even in the lower Mekong region, where the proportion of patients positive on day 3 broadly matches that of isolates with K13 propeller mutations, only certain Kelch propeller domain mutations are associated with higher RSA_0–3hr_ survival rates (44). Mutations in the Kelch propeller domain do not always confer reduced clinical sensitivity to artemisinin (33), and links of *kelch* mutations to artemisinin resistance are dependent on the larger population structure, as detailed by the MalariaGEN Plasmodium falciparum Community Project (45).

### Conclusion.

The current WHO definition of artemisinin resistance rests on two primary indicators: (i) a high prevalence of isolates with a delayed parasite clearance phenotype in a population and (ii) a high prevalence of *kelch* gene mutants (>10% of the population). A confirmed case of artemisinin resistance involves both these components in the same patient (4). In a large and populous country like India, with a high absolute number of cases of malaria but a low prevalence due to the large denominator, considerable resources will be required to perform large-scale clinical assessments of artemisinin resistance across the country. Also, in a study like ours, the size of the sample set is limited by the number of successfully lab-adapted clinical isolates at a time point. So, while the current sample set provides an account of the *in vitro* artemisinin sensitivity in Indian isolates during the study period, it is underpowered to formally infer wider conclusions regarding possible artemisinin resistance in India.

However, the elevated RSA_0–3hr_ survival rate and the concomitant presence of *kelch* mutations in this preliminary sample set, along with clinical reports of delayed parasite clearance from eastern India (14, 15), point toward changing artemisinin sensitivity and warrant wider surveillance of the resistance-associated phenotype and genotype for artemisinin as well as partner drugs in this region.

Finally, our data suggest that the surveillance priority for artemisinin resistance in India should not be determined purely on the basis of the geographic proximity to Southeast Asia or *kelch* mutations alone. A countrywide surveillance of ACT efficacy that incorporates clinical, genotypic, and phenotypic indicators is needed to obtain a complete and accurate picture of possible decreasing artemisinin sensitivity throughout India. The results from the present study justify such strategies and investments.

## MATERIALS AND METHODS

### Ethical statement.

The human subjects protocol and consent forms for enrolling *Plasmodium*-infected patients in this study at Assam Medical College and Hospital (AMC), at Goa Medical College and Hospital (GMC), and at primary health centers (PHCs) in the northeast region were approved by the Institutional Review Boards of the Division of Microbiology and Infectious Diseases (DMID) at the U.S. National Institute of Allergy and Infectious Diseases (approval DMID 11-0074), the University of Washington (approval 42271/1192), as well as AMC, GMC, and the Regional Medical Research Center—Northeast Region (RMRC-NE).

### Sample collection.

Venous blood samples were collected in 6-ml Vacutainer tubes (with acid citrate dextrose solution anticoagulant; BD India) from study participants at AMC in Dibrugarh, GMC in Goa, and PHCs in Arunachal Pradesh, Assam, and Tripura between April 2012 and October 2015. Febrile patients aged between 12 months and 65 years diagnosed with possible malaria by the PHC or hospital were tested for P. falciparum infection by microscopy and a rapid diagnostic test (RDT; FalciVax; Zephyr Biomedicals, Goa, India). Pregnant (self-reported) and anemic patients were excluded from screening by the study team. The initial parasite density and parasitemia (day 0 parasitemia) were determined concurrently at this point. Subsequently, the patients were inducted into the study after appropriate informed consent and prior to the administration of the first ACT dose. The collected samples were processed, cryopreserved in Glycerolyte 57 preservative (Baxter), and stored at –80°C in the MESA-ICEMR facilities at the Regional Medical Research Center (RMRC), Dibrugarh, India, and at GMC.

### *In vitro* culture adaptation.

All the clinical parasite samples were culture adapted to grow under laboratory conditions. Cryopreserved P. falciparum samples were thawed and propagated according to our previously established protocol (46). The patient isolates were grown in 2% hematocrit human type A-positive red blood cells (Rotary Blood Bank, New Delhi, India) suspended in ready-to-use RPMI 1640 medium (catalog number 22400089; Gibco) supplemented with 0.5% AlbuMAX II (Life Technologies) and 0.1 mg/ml hypoxanthine (Sigma-Aldrich). Quality control prior to usage for media and blood was performed by conducting at least two 48-h growth tests with P. falciparum reference line 3D7 or Dd2. No antibiotic was added to the medium to avoid potential confounding changes of the native phenotypes or genotypes of the samples. Culture adaptation of the patient isolates was considered successful after completion of two successful growth tests, each with at least 4-fold growth (46).

### Ring-stage survival assay.

Twenty-two *in vitro* culture-adapted lines were analyzed for artemisinin sensitivity by the ring-stage survival assay (RSA). Twelve of these lines were adapted at RMRC in Assam, and 10 were adapted at GMC in Goa. The collection included samples from patients with high levels of parasitemia as well as patients with low levels of parasitemia. Similarly, patients with severe or uncomplicated malaria were also represented (Table 1). Control parasite lines 3D7 and Dd2 and six Cambodian parasite lines (IPC 3445, 3663, 4884, 4912, 5188, 5202) (5, 20) were obtained from BEI Resources/Malaria Research and Reference Reagent Resource Center (MR4).

RSA was conducted according to a previously published protocol with slight modification (20). For each patient isolate, two rounds of sorbitol (Sigma-Aldrich) synchronization were performed to obtain ring-stage parasites. Percoll enrichment (75%; Sigma-Aldrich) was performed approximately 30 h after the second sorbitol synchronization. The parasites were then placed in a 37°C trigas incubator (90% nitrogen, 5% oxygen, 5% carbon dioxide). After a 3-h incubation, a third and final round of sorbitol synchronization was performed. These synchronized ring-stage parasites were distributed in a 48-well plate at 0.5% to 1% parasitemia and 2% hematocrit in complete RPMI 1640 medium containing 0.5% AlbuMAX II and 2.5% heat-inactivated type A-positive human plasma. Cultures were treated with 700 nM dihydroartemisinin (DHA; Sigma-Aldrich) in dimethyl sulfoxide (DMSO; DHA exposed) or with 0.1% DMSO alone (nonexposed, control) for 6 h. After 6 h, the drug was washed away and both the DHA-exposed and nonexposed controls were resuspended in drug-free medium and incubated for another 66 h. Giemsa-stained thin smears were prepared at 72 h for each of the duplicate DMSO controls, as well as for the 700 nM DHA-treated samples. Each smear was independently analyzed by two microscopists, each of whom counted a total of 10,000 red blood cells per treatment replicate. The growth control threshold was set at 1.5 times (i.e., the parasitemia in the DMSO control was at least 1.5 times higher than the starting parasitemia after 72 h of culture) (47).

### Statistical analysis.

All statistical analyses were completed using GraphPad Prism (version 6) software. The correlation between the two microscopists’ parasitemia counts was determined using the Pearson correlation method. A survival rate threshold was determined using the maximum value (100th percentile) for the negative controls and the minimum value (0th percentile) for the positive controls. Parasite samples were separated into groups using the location of collection (southwest India, northeast India, Cambodia) as the independent variable. These three groups were analyzed by the one-way Kruskal-Wallis test followed by individual Mann-Whitney tests. Findings were considered significant when *P* was <0.05. The threshold survival rate for artemisinin resistance was based on previously reported values, as well as survival trends for the positive and negative controls from the current study.

### *kelch* sequencing.

A portion of all adapted samples was cryopreserved before RSA and subsequently thawed to extract DNA for *kelch* sequencing. Parasite genomic DNA was extracted from highly sorbitol-synchronized ring-stage parasites (>90% rings, 3% to 6% parasitemia) using a Qiagen QIAamp DNA minikit (48). Samples were submitted to Eurofins India (Bangalore, India) for sequencing.

First, the paired-end 100-bp short reads were quality filtered in the TrimGalore and Cutadapt (49) tools to remove all reads that were shorter than 70 bp or that had a quality score below 28. Subsequently, the filtered short reads were processed through our analysis pipeline, which is largely based on the best practices guidelines of the Genome Analysis Toolkit (GATK) (50). Specifically, short reads were aligned to the reference genome (PlasmoDB Pfv9) (51) using BWA-MEM (52). SortSam and MarkDuplicates from PicardTools (https://github.com/broadinstitute/picard) were used to sort the aligned reads and mark the duplicates, respectively. GATK tools were used to realign around indels and recalibrate the quality scores. Mpileup from Samtools (53) was then used to call variants from all samples taken together. This gave a single variant call format (VCF) file that contained all variants from the entire sample set. The SnpEff tool (54) was then used to add annotations to the VCF file. Paired-end 100-bp reads from the sequencing were aligned to the P. falciparum reference genome (PlasmoDB Pfv9). Custom R scripts that leveraged the *VariantAnnotation* package from the Bioconductor suite (55) were used to filter variants on the basis of mapping quality and read depth. Only those which had read depths of greater than 10 and a quality score above 100 were selected. Variants in intergenic regions and on *var* genes were removed. Filtered variants were analyzed using custom R scripts.

### Data availability.

Sequences have been deposited in GenBank under accession no. MK949521 and MK949522.
